# A New Long-Term Composite Drug Delivery System Based on Thermo-Responsive Hydrogel and Nanoclay

**DOI:** 10.3390/nano11010025

**Published:** 2020-12-24

**Authors:** Cezar Tipa, Maria T. Cidade, Tânia Vieira, Jorge Carvalho Silva, Paula I. P. Soares, João Paulo Borges

**Affiliations:** 1 CENIMAT/I3N, Departamento de Ciência dos Materiais, Faculdade de Ciências e Tecnologia, Universidade Nova de Lisboa, 2829-516 Caparica, Portugal; c.tipa@campus.fct.unl.pt; 2 CENIMAT/I3N, Departamento de Física, Faculdade de Ciências e Tecnologia, Universidade Nova de Lisboa, 2829-516 Caparica, Portugal; ts.vieira@fct.unl.pt (T.V.); jcs@fct.unl.pt (J.C.S.)

**Keywords:** biomaterials, long-term drug delivery, injectable hydrogels, nanoclay

## Abstract

Several problems and limitations faced in the treatment of many diseases can be overcome by using controlled drug delivery systems (DDS), where the active compound is transported to the target site, minimizing undesirable side effects. In situ-forming hydrogels that can be injected as viscous liquids and jellify under physiological conditions and biocompatible clay nanoparticles have been used in DDS development. In this work, polymer–clay composites based on Pluronics (F127 and F68) and nanoclays were developed, aiming at a biocompatible and injectable system for long-term controlled delivery of methylene blue (MB) as a model drug. MB release from the systems produced was carried out at 37 °C in a pH 7.4 medium. The Pluronic formulation selected (F127/F68 18/2 wt.%) displayed a sol/gel transition at approx. 30 °C, needing a 2.5 N force to be injected at 25 °C. The addition of 2 wt.% of Na116 clay decreased the sol/gel transition to 28 °C and significantly enhanced its viscoelastic modulus. The most suitable DDS for long-term application was the Na116-MB hybrid from which, after 15 days, only 3% of the encapsulated MB was released. The system developed in this work proved to be injectable, with a long-term drug delivery profile up to 45 days.

## 1. Introduction

One significant problem faced in the treatment of many diseases is the poor delivery of therapeutic compounds to the target site and undesired collateral effects. Treating a disease with multiple dosing strategies and using conventional drug formulations has many drawbacks such as fluctuation in drug plasma concentration, ineffective treatments, or even various toxic side effects [1,2]. This is due to poor biodistribution, limited effectiveness, and lack of selectivity of conventional drug delivery. An effective treatment should maintain the drug plasma level within the therapeutic concentration range for as long as the treatment is required [1]. This can be attained by controlled and targeted drug delivery systems (DDS) where the active compound is transported to the target site and its influence on healthy tissues and undesirable side effects are minimized. Additionally, DDS protects the drug from rapid degradation and enhances its effective impact on targeted tissues for more extended periods than conventional delivery with favorable release kinetics, thus requiring lower doses and less frequent administration [3]. Several studies have addressed the possibility of implementing long-acting treatments, aiming at reducing the medication-related burden and increasing adherence and treatment efficiency in different therapeutic areas such as opioid use disorders [4], contraception [5,6,7,8], to overcome tumor drug resistance [9], antipsychotic therapy [10], cancer treatment [11], macular degeneration [12], delivery of antimicrobial substances deposited on medical implanted materials [13], inflammation control [14], and chronic diseases [15], among others.

The recent development in polymer chemistry and biomaterials science has been focusing on in situ-forming hydrogels that can be injected as viscous liquids and jellify under exposure to the physiological environment [16,17]. Hydrogels are entangled three-dimensional (3D) polymer networks with a high number of hydrophilic groups with a high affinity for water. These biomaterials have gained attention due to their swelling, drug protection in in vivo environments and sensitivity to different stimuli, among others [18]. Thermo-sensitive polymers like poloxamers (trade name Pluronic) are synthetic nonionic compounds with an ABA-type triblock copolymer structure. Pluronic F127 (Poloxamer P407) is one of the most widely used to prepare thermosensitive hydrogels for drug delivery, being approved by the FDA and considered non-toxic [19]. F127 hydrogels have been studied as topical drug delivery carriers by different administration routes such as subcutaneous and intramuscular [19]. These polymers are formed by a central hydrophobic group of polypropylene oxide (PPO), flanked by two hydrophilic polyethylene oxide blocks (PEO) (PEOx-PPOy-PEOx) [20,21]. Pluronic hydrogels have a lower critical solution temperature (LCST), above which the hydrogel is formed [1,22,23,24]. This transition is a reversible process that occurs at a temperature depending on the type of Pluronic involved (i.e., the chemical structure with different PEO and PPO group lengths and ratios) and its concentration in an aqueous solution. However, even if Pluronic spontaneously forms a gel above a specific LCST, its structural integrity is not maintained for a long time in the physiological environment. Its low molecular weight and low mechanical strength results in premature hydrogel dissolution and burst drug release, limiting its applications [19,21]. To address this problem, researchers have combined the poloxamer with other materials and reinforcements. Clay reinforcement offers a possible solution to enhance the Pluronic hydrogels’ structural integrity, control drug release kinetics, and optimize its bioactivity and therapeutic effect [2]. Clay nanoparticles are two-dimensional layered materials with non-toxic degradation byproducts and present beneficial properties for osteogenic cell function, among others [25]. Since the amount of clay loading required to obtain significant variations in the polymer matrix is very low, aside from being an abundant and low-cost material, this association is of great interest.

Montmorillonite (MMT), the major constituent of bentonite, is one of the most widely used clay minerals in the fabrication of polymer–clay nanocomposites (PCNs) [26,27]. It belongs to the smectite group, and is composed of silica tetrahedral sheets between alumina octahedral sheets, which originates from the structural family of a 2:1 clay layer, also called phyllosilicates [26]. Montmorillonite shows extensive interlayer expansion when water or other polar molecules intercalate into their layers, high specific surface area, and high cation exchange capacity. One important consequence of the charged nature of clays is their high hydrophilicity, which makes them incompatible with a wide range of organic and non-polar polymers [27,28]. To make it compatible with organic polymers, the clay’s surface should be modified to be organophilic before use. This is important to weaken the interlayer cohesive energy and promote a more compatible polymer–clay (organic–inorganic) interaction. Organophilic clay can be obtained by a cation exchange reaction of the hydrophilic clay with an organic cation such as alkylammonium. The inorganic and relatively small ions (interlayer Na^+^ and others) are exchanged with these organic salts. This organic modification has two consequences: the expansion of the interlayer distance with a d-spacing increase (typically > 2 nm), enabling organic polymer chains to move in between them, and the change from hydrophilic to hydrophobic or organophilic of the surface properties of every single sheet [27]. Alkyl ammonium ions also reduce the electrostatic interactions between the silicate layers, facilitating the penetration of the polymer into the galleries of the MMT. Depending on the type of alkyl ammonium used, the structure and final properties of the nanoclay can differ widely. Currently, both pristine and organo-modified MMT (OMMT) are available with the commercial name Cloisite [25,29].

Encapsulating the drug into the nanoclays’ interlaminar space might be an effective strategy to address the challenge of prolonged dosing regimens and subsequently to enhance therapeutic efficiency of the bioactive compounds by keeping the active agent concentration in the local targeted environment within the therapeutic range, as long as the treatment requires, as recently studied [30,31,32,33,34]. The challenge in designing an ideal long-term drug delivery system is to balance the ease and safety of administration with the adjustment/modeling of the drug release rate at the appropriate concentration to obtain the desired long-term therapeutic effect [3]. This study reports the development of an injectable composite DDS based on a thermosensitive hydrogel (Pluronic) and nanoclay. The two main objectives were to demonstrate its capacity to be administered through parenteral routes, safely hold a drug load, and provide a controlled drug release over several weeks. The minimally invasive long-term DDS developed in this study may be used for various parenteral applications such as management/treatment of chronic conditions wherein long-term drug therapy is needed.

## 2. Materials and Methods

### 2.1. Materials

Ultrapure water (Míli-Q, Merck, Darmstadt, Germany), ethanol (Sigma-Aldrich, 99.8%, St. Louis, MO, USA), and acetone (Fisher Chemical, Hampton, NH, USA) were used as solvents. For the preparation of Pluronic formulations, Pluronic F127 (Poloxamer P407, EO_98_PO_69_EO_98_, Mw 12,000 Da) and Pluronic F68 (Poloxamer Kolliphor P188, EO_80_PO_27_EO_80_, Mw 8400 Da) copolymers (Sigma-Aldrich, %, St. Louis, MO, USA) were used. Natural montmorillonite (Nanofill 116) and several commercial organically modified montmorillonites (Cloisite 10A, Cloisite 15A, Cloisite 20, Cloisite 30B, and Cloisite 93) from Rockwood Clay Additives were used in this study without purification (Appendix A). High purity methylene blue (MB, Alfa Aesar, Haverhill, MA, USA) was used as a model drug for drug delivery studies. Additionally, for drug delivery studies, a phosphate buffer saline with pH 7.4 (PBS 7.4) solution was prepared with the following precursors: sodium chloride (NaCl), potassium chloride (KCl), disodium phosphate (Na_2_HPO_4_), and potassium dihydrogen phosphate (KH_2_PO_4_,) all from Sigma-Aldrich (St. Louis, MO, USA).

### 2.2. Pluronic Formulations

Pluronic formulations were prepared by dissolving the required amounts of Pluronic F127 and F68 in cold water, according to the previously described cold method, and left at 4 °C until a transparent/clear solution is formed (one to two days) [35]. Pluronic F127 solutions were prepared with concentrations ranging from 15 to 25 wt.%, while the Pluronic F68 solution was prepared with a 20 wt.% concentration. Mixtures of Pluronic F127 and F68 were also prepared with the following weight ratios: 19/1, 18/2, 17/3, and 16/4.

### 2.3. Pluronic–Clay Composites and Drug-Loaded Clays

For Pluronic–clay composites, nanoclay particles were simply added to the Pluronic formulation under magnetic stirring at 4 °C (ice bath) until a homogeneous suspension was obtained. To prepare methylene blue intercalated clays (clay-MB hybrids), a 2 wt.% aqueous clay suspension was first prepared under magnetic stirring in the appropriate solvent for 12 h, followed by 30 min sonication (if required), and another 12 h left alone to swell and expand. Then, a determined pre-dissolved (in the same solvent) amount of MB (depending on the pretended clay to MB ratio) was slowly added to the clay suspension at room temperature, followed by 24 h mixture stirring. Using clays Na116 and C10A, three weight ratios were tested: 20:1, 4:1, and 2:1. Given the results obtained, the remaining clays were mixed with MB using a 2:1 weight ratio. At the end of the process, the clay-MB hybrid’s/MB-loaded clay’s solid phase was separated from the liquid phase by centrifugation using a Heraeus Multifuge X1R Centrifuge from Thermo Fisher for 20 min at 10,000 rpm. The solid pellet phase was washed a couple of times. The centrifugation resultant clay-MB pellet was freeze-dried (VaCo 2, Zirbus Technology) or dried at room temperature [36].

### 2.4. Characterization Methods

X-Ray Diffraction (XRD) measurements were performed on X’Pert PRO PANAlytical diffractometer (Malvern, UK), in the range 1° < 2θ < 10° to determine the basal spacing of the clay powders. A Cu Kα radiation, generated at 40 kV and 40 mA was used. Fourier Transform Infrared Spectroscopy (FTIR) spectra of the clays were obtained using a Nicolet 6700-Thermo Electron Corporation Attenuated Total Reflectance-Fourier Transform Infrared spectrometer (ATR-FTIR, Madison, WI, USA) with a resolution of 2 cm^−1^, in the 500–4000 cm^−1^ wavenumber range.

To evaluate methylene blue drug loading and encapsulation efficiency by intercalation or adsorption onto clays, UV–VIS (for the supernatant MB solution) measurements were made [13]. In all experiments using methylene blue, its concentration in solution was determined by UV–VIS (UV–Vis, T90+ PG Instruments, Leicestershire, UK). Concentration was calculated from calibration curves obtained from absorbance measurements of solutions with known MB concentrations. The absorbance was measured in the wavelength of 664 nm, corresponding to the MB maximum absorbance peak. MB encapsulation efficiency into clays and resultant nanocomposite properties were tested for different MB to clay ratios and the different clays being studied.

Differential Scanning Calorimetry (DSC) experiments were carried out with a NETZSCH DSC 204 F1 Phoenix (Selb, Germany), in a N_2_ atmosphere. A temperature ramp was performed at 1 °C/min from 5 °C until 50 °C. The Pluronic’s micelle formation temperature was determined from the endothermic peak in the recorded DSC thermograms. In clays, the degradation/decomposition and weight loss stages were evaluated as a function of temperature. The heating rate was 10 °C/min, from room temperature to 900 °C in a N_2_ atmosphere.

Rheological characterization: Rheological measurements of Pluronic formulations and composite formulations were performed with a Modular Compact Rheometer MCR 502 (Anton Paar, Madrid, Spain), using a parallel-plate geometry (diameter 25 mm) with a 1 mm gap. Oscillatory (dynamic) and stationary measurements were carried out. For oscillatory experiments, temperature ramps were performed, where storage G’ and loss G” moduli were measured under a constant strain of 0.5%, within the linear viscoelastic regime in which the moduli are independent of the strain, and a frequency of 1 Hz. The temperature ramp was carried out from 15 °C to 50 °C at 1 °C/min rate. Isothermal measurements were also carried out at selected temperatures, G’ and G” being recorded as a function of time, at an angular frequency of 1 Hz and 1% strain. Additionally, to measure the viscosity as a function of the shear rate, between 1 and 1000 s^−1^, stationary measurements at temperatures representative of surgery room temperature were performed.

Injectability tests: For the injectability tests, Pluronic-clay composites were injected from a 3 mL syringe, with a compression speed of 4 mm/s, with the aid of a 3D-printed support for the syringe and a universal compression testing machine (Autograph AG, Shimadzu, Kyoto, Japan).

### 2.5. Encapsulation Efficiency

To evaluate methylene blue drug loading and encapsulation efficiency by intercalation or adsorption onto clays, DSC-TGA (Differential Scanning Calorimetry and Thermogravimetric analysis for the nanocomposite powder) and UV–VIS (for the supernatant MB solution) measurements were made [13]. In all experiments using methylene blue, its concentration in solutions was determined by UV–VIS (UV–Vis, T90+ PG Instruments). Concentration was calculated from calibration curves obtained from absorbance measurements of solutions with known MB concentrations. The absorbance was measured in the wavelength of 664 nm, corresponding to the MB maximum absorbance peak. MB encapsulation efficiency into clays and resultant nanocomposite properties was tested for different MB to clay ratios and the different clays being studied.

### 2.6. Methylene Blue (MB) Release Studies

In vitro MB drug delivery profiles were determined in PBS with pH 7.4 at 37 °C, simulating physiological conditions. The system used for drug release measurements comprised two compartments, a donor and a receiver, connected through a permeable membrane. For this purpose, the developed drug delivery systems were added to the donor compartment (3 mL system) and 50 mL of PBS7.4 were added to the receiver compartment. The whole system was kept at a constant temperature of 37 °C while magnetically stirred (approx. 60 rpm). At regular periods, 25 mL of the receiver release medium were removed and replaced by 25 mL of fresh PBS. Three mL of the withdrawn release medium were analyzed by UV–VIS spectroscopy to determine the MB concentration.

### 2.7. Cytotoxicity Assays

To evaluate the cytotoxicity of the produced systems, the tests were performed according to standard ISO-10993 Biological evaluation of medical devices, Part 5: Tests for in vitro cytotoxicity. The assays were performed using the extract method and Vero cells. To determine the number of viable cells, a colorimetric method based on resazurin and its reduction to resorufin by live cells was used. As the absorption peak of resazurin shifts from 600 nm to 570 nm upon reduction, absorbance measurements allow the quantification of the number of active cells relative to the negative cell control, and consequently the assessment of the cytotoxicity of the material being studied. The negative control corresponds to cells fed with a complete cell culture medium and the positive control corresponds to cells cultured in a cytotoxic environment caused by the supplementation of the culture medium with 10% dimethyl sulfoxide. The culture medium consisted of DMEM (Dulbecco′s modified Eagle′s medium with 1.0 g/L glucose, with stable glutamine, with sodium pyruvate, Biowest #L0066) supplemented with penicillin (100 U/mL) and streptomycin (100 µg/mL) (Invitrogen, #15140122) and 10% FBS (Fetal Bovine Serum, S. America origin, Biowest, #S1810).

## 3. Results

The main purpose of this study was to produce an injectable composite DDS using a thermo-sensitive hydrogel based on Pluronic and nanoclay. The first part of the work focused on hydrogel optimization to obtain an injectable system. In this case, the gelation temperature (Tgel) of Pluronic formulations are considered suitable between room (25 °C) and human body temperature (37 °C). If the gelation occurs at a temperature higher than 37 °C, the hydrogel remains in its solution state when administrated into the physiological medium. Next, MMT and OMMT were fully characterized and incorporated into the optimal Pluronic formulation. Before drug release studies using methylene blue as a model drug, preliminary characterization was performed to evaluate and select the most suitable composite formulations for long-term drug release.

### 3.1. Pluronic Formulation

The temperature-responsive properties of Pluronic hydrogels are generally evaluated by the sol/gel transition temperature determination. This information can be obtained with temperature ramps in oscillatory shear mode by identifying the temperature at which storage modulus G’ and loss modulus G” undergo critical variations. The higher the G’ value, the more pronounced the elastic properties, and the higher the G”, the more pronounced the viscous properties [37]. The elastic modulus is a measure of the energy stored and reflects the solid-like component or elastic behavior. The sol/gel transition temperature is usually defined as the intersection of the G’ and G” moduli [38].

**Figure 1:**
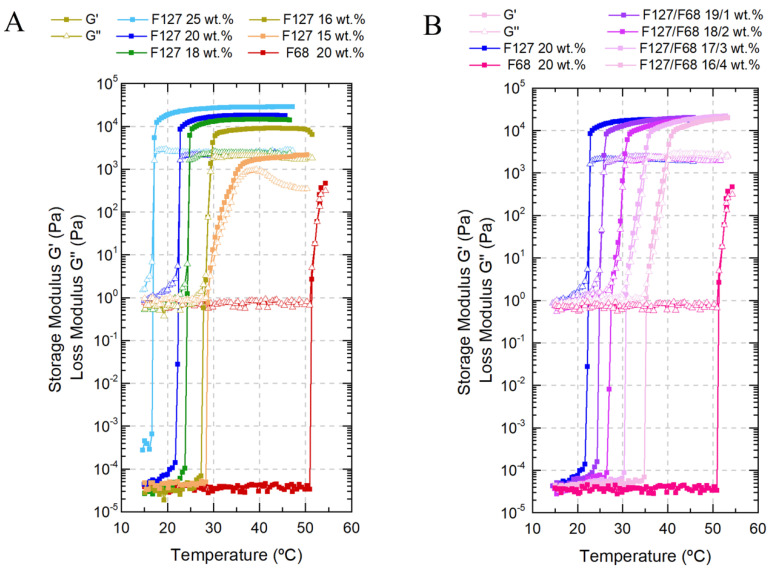
Viscoelastic behavior of all Pluronic formulations as a function of temperature: (**A**) single F127 formulation with concentrations ranging from 15 wt.% to 25 wt.%, and F68 formulation with a concentration of 20 wt.%; (**B**) binary F127/F68 with weight ratios from 20/0 (F127 20 wt.%) to 16/4 (F127/F68 16/4wt.%).

Single F127 hydrogels (Figure 1A) were tested with increasing concentrations from 15 wt.% to 25 wt.%, leading to an increase in their storage modulus G’ and therefore enhanced structural integrity and elastic properties, with a decrease in the gelation temperatures from 29 °C to 17 °C. Above 16 wt.% concentration, in the gel state, the loss modulus G” did not suffer significant changes, which means that all of the gels maintained similar viscous properties. Single F68 hydrogels (Figure 1A) at 20 wt.% displayed a viscoelastic transition at approximately 51 °C, although some studies have reported gelation at higher temperatures (approx. 60 °C) [39].

To properly adjust the transition temperature to produce an in situ gelling system, F127/F68 binary mixtures (Figure 1B) were tested. The F68 addition effect on the Tgel was evident, changing Tgel from 22 °C to 36 °C (by approx. +14 °C) when the F127 concentration decreased from 20 to 16 wt.% and F68 increased from 0 to 4 wt.%, respectively. The addition of F68 resulted in a continuous increase of the storage modulus, even after gelation. The sol/gel transition became less spontaneous and more delayed compared to single F127 solutions. The viscous contribution (due to slightly higher loss modulus with F68 increase) is likely related to the non-crystalline phase mainly composed of the F68 polymeric chains or micelles [35,38].

**Figure 2:**
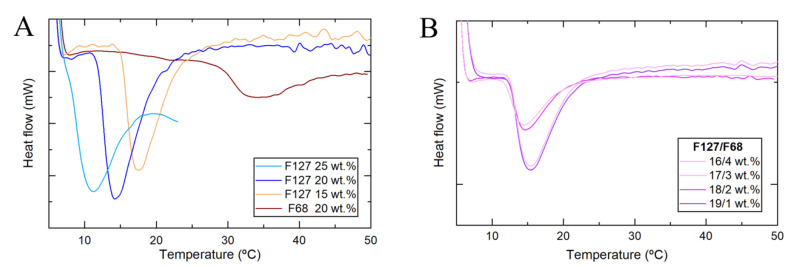
Micellization behavior of single (**A**) and binary (**B**) Pluronic solutions as a function of temperature obtained from differential Scanning Calorimetry (DSC) measurements.

To better understand the micellization process, DSC experiments were done. The broader peaks corresponded to the progressive formation of Pluronic micelles in the formulation with increasing temperature [35]. The micellization formation is endothermic and can be analyzed by the onset temperature (T_onset_), the peak temperature (T_peak_), and the endset temperature (T_endset_) [38,40]. Different concentrations of F127 and F68 hydrogels, alone (Figure 2A) or mixed (Figure 2B) with a fixed total Pluronic concentration of 20 wt.% were analyzed. For the single F127 formulations, variation in their concentration changed the micellization temperatures. The onset temperature at the concentration of 25 wt.% was 8.2 °C, which is only approx. 3 °C above the dissolution temperature, meaning that the micellization temperature is close to this solution solubility limit [37,38,40,41]. The plain solution of F68 at 20 wt.% shows a clear endothermic peak due to micelle formation. The micellization peak for F68 was smaller than F127 at the same concentration and was broader in temperature (Figure 2A). This broader micellization peak can explain the continuous rheological increase in the elastic modulus of F127/F68 formulations, even after gelation. F127/F68 mixtures (19/1, 18/2, 17/3, and 16/4 wt.%) had similar micellization behavior to the solution containing 20 wt.% F127 (Figure 2B). Only one peak of micellization was observed for these systems, indicating that only F127 was able to form micelles because, in these conditions, F68 had concentrations lower than the critical micelle concentration (CMC) or the enthalpic contribution on micellization was too small to be detected, as previously described in the literature [38]. In Table 1, the characteristic temperatures obtained from DSC (T_onset_, T_peak_, T_endset_) and the rheological experiments of all Pluronic formulations are summarized.

**Figure 3:**
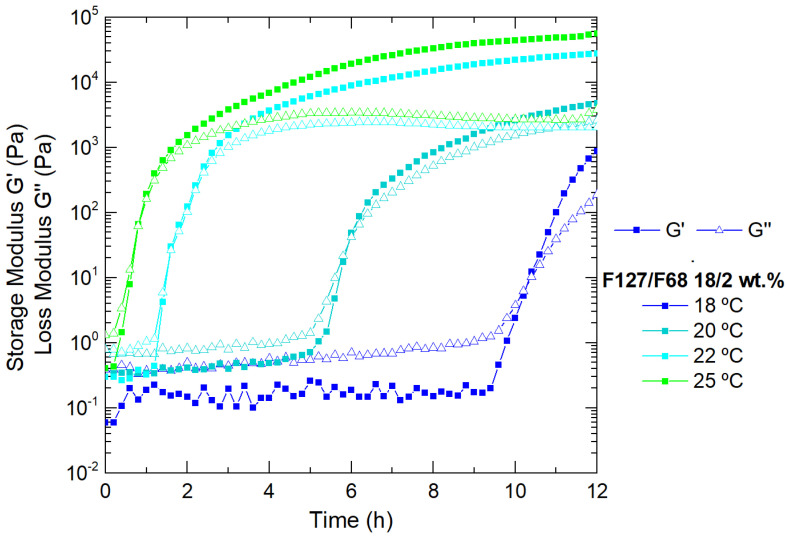
Viscoelastic behavior of the selected Pluronic formulation (F127/F68 18/2 wt.%) as a function of time, at a fixed temperature (18 °C, 20 °C, 22 °C, and 25 °C).

Further characterization and development of our drug delivery system were performed using the selected Pluronic binary mixture: F127/F68 18/2 wt.%, due to its sufficiently high viscoelastic modulus and its sol/gel transition temperature at approximately 28 °C, which best suits our objective of an injectable in situ gelation system. Isothermal measurements (Figure 3) were made to analyze the selected binary system (F127/F68 18/2 wt.%) at fixed temperatures for long periods to evaluate its rheological and structural behaviors before injection. The experiments were made at T < Tgel. It is clear that approaching the gelation temperature (28 °C), the solution contains large amounts of micelles, but not yet in the gel state (packed crystalized structure), evolving toward more elastic systems [38]. Initially, G’ < G” in this range of temperatures, and both moduli increased slowly with time, but after a few hours, depending on the respective temperature and what it entails in terms of the number of micelles at that temperature, the storage modulus overtook the loss modulus and increased continuously until the number of micelles formed at that temperature were fully packed. The increase of the moduli at this frequency was over more than a decade, which was a quite significant effect. If the temperature is not higher than the endset temperature of the micellization process, the moduli increase will never reach the level of those, for which all the micelles are fully formed. These results indicate that the system is not fully stabilized after 12 h. There is a slow structuring in the solution at a fixed temperature, which may be interpreted as a local ordering of the micelles toward the formation of emerging local crystalline arrangements [38].

**Figure 4:**
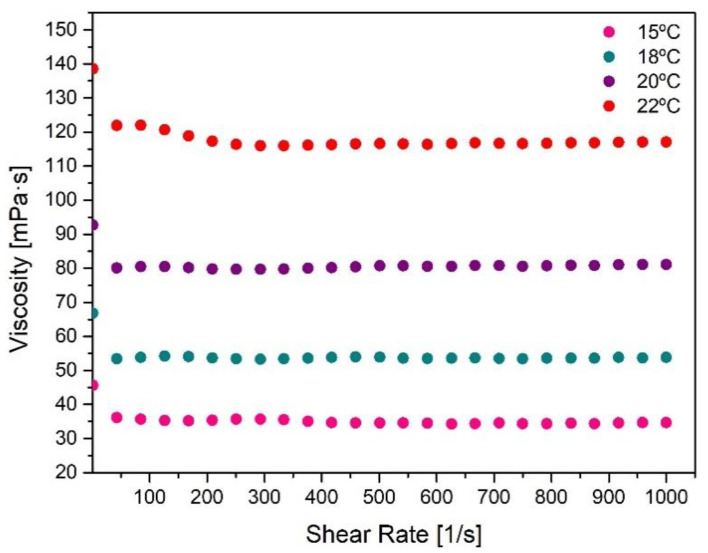
Flowability curves of the selected Pluronic formulation (F127/F68 18/2 wt.%) as a function of shear rate, at fixed temperatures (15 °C, 18 °C, 20 °C, and 22 °C).

The behavior of the selected F127/F68 binary mixture during injection was also studied through flowability curves as a function of shear rate (Figure 4). When the system is injected through a narrow device like a syringe, a sufficiently low viscosity is necessary to minimize the flow resistance through the syringe as well as high shear rate invulnerability, thus maintaining its structure and properties. It is possible to verify that the Pluronic F127/F68 formulation viscosity does not suffer significant changes with increasing shear rates. However, as the temperature increases, its viscosity also increases, which can be explained by approximating the gelation temperature. Even though an abrupt variation of the elastic modulus can be observed when the LCST/Tgel is reached, viscosity changes also occur before this sol/gel transition due to the micellization process, explaining the increasing viscosity before gelation [35,38,40].

### 3.2. Clay Characterization

**Figure 5:**
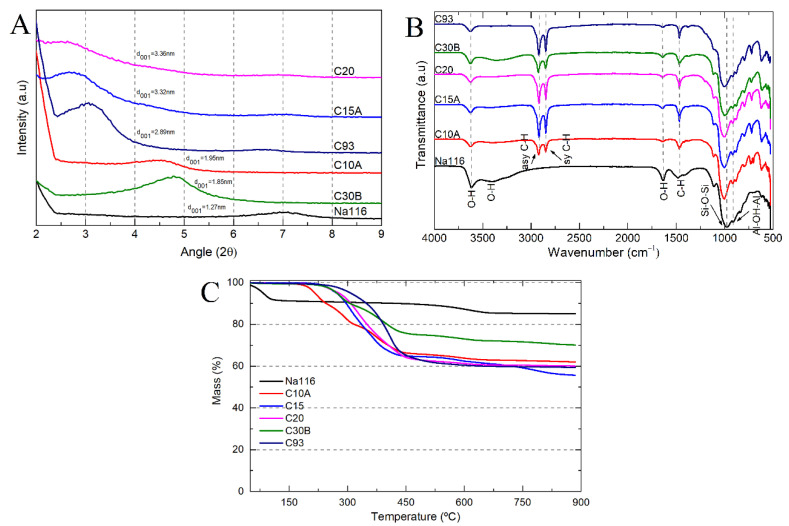
(**A**) Clay basal spacing obtained from x-ray diffraction (XRD) measurements; (**B**) clay Fourier transform infrared (FTIR) spectra; and (**C**) clay degradation stages as a function of temperature.

Based on the commercially available clays, six samples were selected: unmodified montmorillonite (Na116) and five organically modified MMT (C10A, C15A, C20, C30B, and C93). All clays were characterized by XRD, FTIR, and TGA to evaluate the differences between them. XRD is one of the most important characterization techniques for assessing the changes in the clays’ basal spacing (d_001_), thus identifying whether the MMT has been successfully modified or not. The expansion of the clay basal spacing is evidenced by shifting the diffraction peaks toward smaller angles [27,42]. The clay basal spacing (d_001_) was verified by XRD measurements (Figure 5A), demonstrating that different organic modifiers lead to different basal spacing for the OMMT clays.

Due to its sensitivity in detecting organic functional groups, FTIR was used to determine the chemical interaction of the organic modifiers with the MMT (Figure 5B). All spectra showed bands at 3636 and 3395 cm^−1^ attributed to the structural O–H stretching vibrations of the aluminum/silicate/magnesium in MMT and adsorbed water, respectively. Bands at 1640 and 1475 cm^−1^ (O–H bending of adsorbed water and C–H bending), a strong band at 1002 cm^−1^ (stretching vibration of Si–O–Si from silicate), and a band at 917 cm^−1^ (Al–OH–Al deformation of aluminates) can also be seen. In OMMT’s spectra, new bands were located at 2924 and 2842 cm^−1^, attributed to the vibration of C–H methylene groups (symmetric stretching and asymmetric stretching, respectively). The peaks corresponding to adsorbed water (3395 cm^−1^ and 1640 cm^−1^) were less visible in the OMMT spectra than in the MMT one, which may be due to the hydrophobic character of OMMT clays. Some differences could be seen in the intensities of the 2924, 2842, and 1475 cm^−1^ bands, probably due to differences in the amount and type of modifier content present in the OMMT clays. The C30B clay had a broader and more prominent band at 3395 cm^−1^ than all the other OMMT, attributed to the O–H stretching due to bis-2-hydroxyethyl groups present in the organic modifier [43].

The weight loss as a function of temperature can be used to determine the amount of organic modifiers present in the nanoclay. The thermograms of the unmodified MMT and OMMT can be seen in Figure 5C. In both MMT and OMMT, the initial weight loss below 200 °C was attributed to the evaporation of physically adsorbed water. OMMT showed a lower free water content than MMT due to the intercalation of organic modifiers that increases the clay’s hydrophobicity. The weight loss between 200 and 600 °C was attributed to the degradation of adsorbed or chemically linked (grafted) modifiers from the surface of the OMMT. The amount of grafted or adsorbed modifier on the clay surfaces can be calculated based on the percentage of lost weight between 200 °C and 600 °C. The weight loss above 600 °C is attributed to the depletion of structural hydroxyl groups and of structural aluminosilicate, after surface modification. The weight loss in this region for OMMT increased considerably compared to pristine MMT, thus confirming the viability of organophilization [26,27]. Table 2 summarizes the basal spacing, water content, and amount of organic modifier within each clay (calculated as the mass loss between 200 and 900 °C, instead of 600 °C). In general, it can be considered that the residual mass is the inorganic material that remained. The results show that the amount of cation modifier on the total mass of clay was high (between 29% and 44% by weight) [42,44].

### 3.3. Pluronic–Clay Composites Characterization

**Figure 6:**
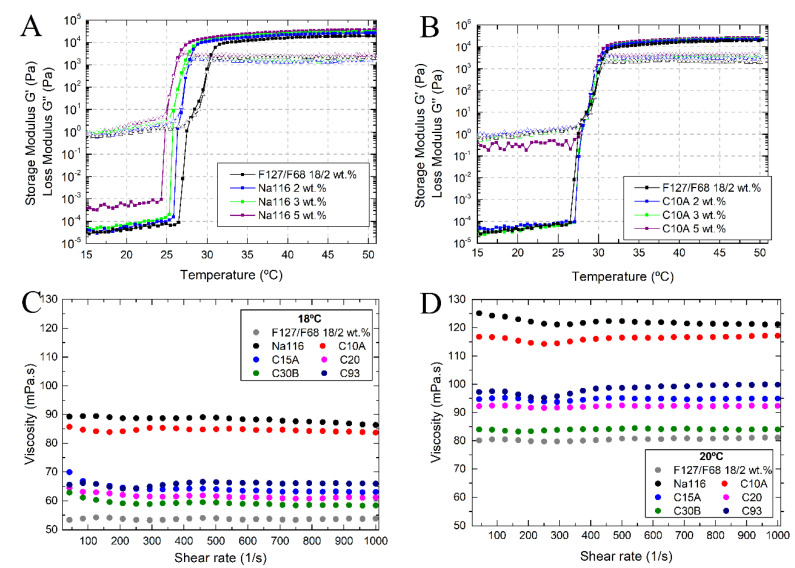
Viscoelastic behavior of Pluronic–clay composites, with Na116 (**A**) and C10A (**B**) clay concentration of 5, 3, and 2 wt.%, as a function of temperature. (**C**,**D**) Viscosity/Flowability curves of Pluronic–clay composites, with clay concentration at 2 wt.%, as a function of shear rate, at temperatures of 18 °C and 20 °C, respectively.

MMT and OMMT clays were incorporated into the selected mixture of Pluronic F127/68 18/2 wt.%. Rheological characterization and injectability tests were performed to evaluate the clay’s effect on the selected F127/F68 binary mixture. Figure 6A,B displays the temperature ramps of the selected Pluronic formulation (F127/F68 18/2 wt.%) mixed with Na116 or C10A clay at 5 wt.%, 3 wt.%, and 2 wt.%, respectively. It is possible to verify that increasing the amount of Na116 added leads to a decrease in the sol/gel transition temperature and an increase in the storage modulus (elastic properties). In C10A, increasing clay amount displayed higher viscoelastic modulus, but no observable changes in the sol/gel transition temperature. Therefore, and since 2 wt.% of Na116 and C10A clay were enough to enhance the Pluronic hydrogel’s structural integrity, this amount was tested for the remaining clays, as shown in Table 3.

Na116 incorporation significantly improved the storage modulus compared to other clays and decreased the loss modulus (viscous properties) compared with plain hydrogel, thus improving the structural integrity of the gel. The addition of OMMT clay showed a small increase in the storage modulus, but a significant increase in the loss modulus, contributing to a more viscous gel. In terms of sol/gel transition, all polymer–clay composites continued above the minimum required of 25 °C for an injectable gelling system.

To evaluate the effect of clay addition on the injectability of the selected Pluronic formulation, the composites’ viscosity/flowability was tested as a function of shear rate at 18 °C and 20 °C (temperatures near the ones found in a surgical room). At 18 °C (Figure 6B), the Na116 and C10A clay-based composites displayed higher viscosity. The viscosity of all composites is practically independent of the shear rate, although some minor variations can occur for smaller shear rates until an equilibrium behavior is reached. This may be, to some extent, due to some composite heterogeneities in clay dispersion. By increasing the temperature from 18 °C to 20 °C (Figure 6C), a consistent increase in their viscosities was observed.

**Figure 7:**
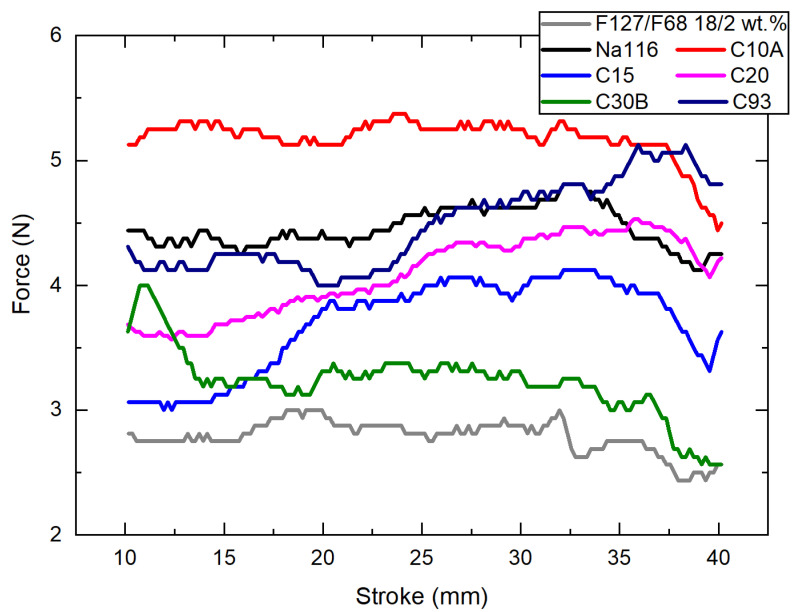
Injectability force required for all Pluronic–clay composites, with a clay concentration at 2 wt.% to be injected from a 3 mL syringe at 4 mm/s at 25 °C.

To evaluate the applicability of the developed composite system as an injectable hydrogel, injectability measurements at 25 °C were performed (Figure 7). The curve irregularities throughout the measure can be explained by air bubbles present in the syringe due to improper filling. The abrupt changes in the force values may be explained by clay heterogeneities or clay aggregates in the injected composites. The needed force to inject these composite systems is within the standard range values required for a system to be considered injectable by a human hand [44], making it a viable system for the envisaged application.

### 3.4. Final Composite System

#### 3.4.1. Methylene Blue Encapsulation Efficiency

**Figure 8:**
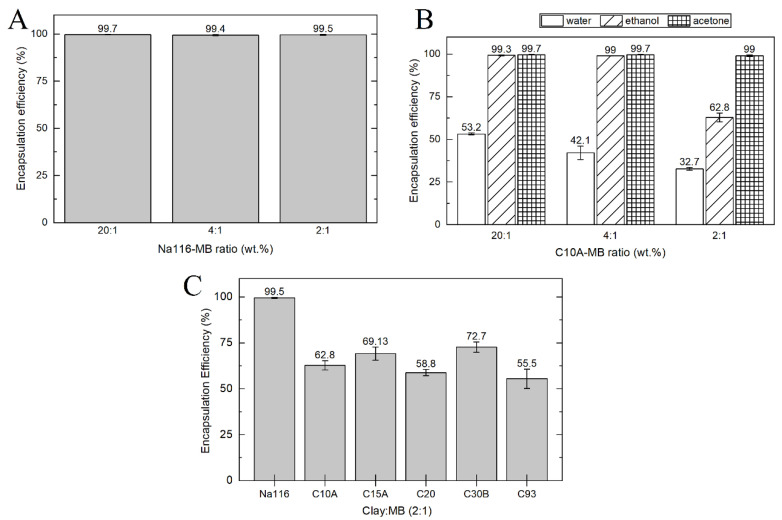
MB EE for (**A**) different Na116-MB weight ratios, prepared in water; (**B**) different C10A-MB weight ratios prepared in different solvents without sonication and (**C**) all clay–MB in a 2:1 ratio prepared in water (Na116) and ethanol (OMMT).

Methylene blue was used as a model drug to evaluate the drug release profile from the clays and composite systems. MB encapsulation efficiency (EE) was calculated by an indirect method using the MB supernatant following encapsulation. The amount of MB was measured by UV–VIS spectroscopy using a previously determined calibration curve. Encapsulation efficiency (EE) was evaluated in different clays, different solvents, and different clay to MB weight ratios. Figure 8A shows Na116 clay encapsulation efficiency, with a higher EE in water, contrary to the EE of C10A (Figure 8B). The weight ratio of clay:MB did not affect the EE on MMT. In Na116 clay, a maximum EE of 99.5% was achieved with a clay:MB weight ratio of 2:1, which indicates that a clay amount of 100 mg can incorporate about 49.75 mg of MB. However, on C10A, the EE decreased with the 2:1 ratio in water and ethanol (EE of 62.8%, which translates into a maximum of 31.4 mg of MB incorporated in 100 mg of clay). This may be due to the hydrophilic character of the MMT surface. Since the incorporation of organic modifiers improves the hydrophobicity of the OMMT and decreases the EE in water, acetone and ethanol were tested as solvents for the C10A clay (Figure 8B) [45,46]. Both solvents displayed better EE than water. To evaluate the EE of MB in remaining OMMT, we used a weight ratio of 2:1. Figure 8C displays the MB EE of all clays tested in a clay-MB hybrid with a 2:1 ratio (MMT prepared in water and OMMT prepared in ethanol). All the following characterization tests for all clays were performed for the 2:1 weight ratio hybrid prepared in water (MMT) or ethanol (OMMT).

#### 3.4.2. Final Composite Characterization

**Figure 9:**
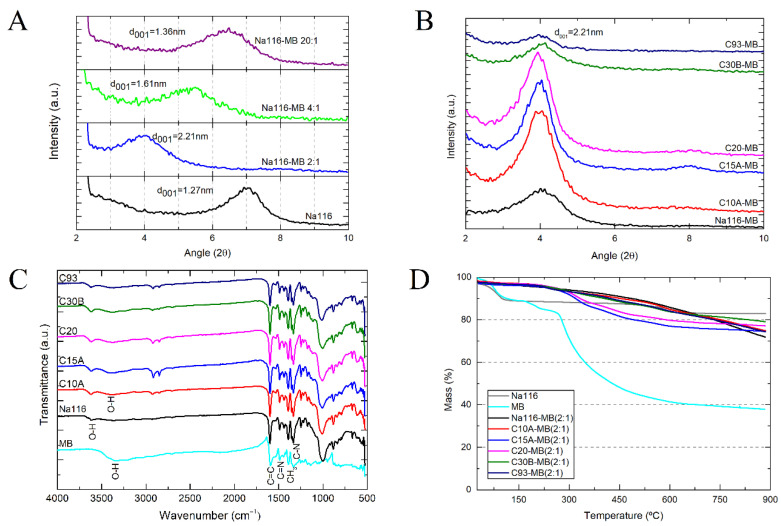
Clay’s final basal spacing after MB encapsulation: Na116 clay:MB weight ratio from 2:1 to 20:1 prepared in water (**A**), and all OMMT clays with a weight ratio of 2:1 prepared in ethanol (**B**,**C**) MB and clay’s FTIR spectra after MB encapsulation, with a clay:MB weight ratio of 2:1, prepared in water for Na116 clay and ethanol for all OMMT clay; (**D**) Clay–MB hybrid degradation stages as a function of temperature.

The MB effect on the clay’s basal spacing (d_001_) was determined by XRD measurements (Figure 9A). It was observed that the XRD peaks for Na116-MB shifted to lower angles, which can be attributed to the intercalation of MB molecules into the interlayer spacing of clay. By increasing MB concentration, the distance between the aluminosilicate layers increased accordingly. These results align with previous studies where higher agglomerates of MB lead to higher MB cation content in the interlayer space, especially for unmodified MMT [36]. All clays intercalated with MB displayed a d_001_ of approximately 2.21 nm (Figure 9B). In MMT and OMMT clays C10A and C30B, the interlaminar space increased because of MB intercalation. However, in the remaining OMMT clays, the interlaminar space decreased. One possible explanation is that for the Na116 clay, MB molecules switch places (by cation exchange reaction) with approximately 6 wt.% of Na cations (present between Na116 interlayers), with the remaining MB molecules increasing the clay’s basal spacing. However, for these OMMT clays, the organic modifiers present between the clay’s interlaminar space were approximately 30 to 40 wt.%, which might not be replenished or surpassed by the MB amount that is intercalated/encapsulated, resulting in a decrease of the basal spacing.

Infrared spectra of MB and all clay–MB hybrids are shown in Figure 9C. All peaks observed in the FTIR spectra of clays appeared on the spectrum of the respective clay–MB hybrids, even though the intensities decreased (due to the cation exchange process between organic modifiers and MB molecules), and small shifts had occurred. It must be mentioned that the FTIR spectrum of MB at wavenumbers between 3000 cm^−1^ and 3500 cm^−1^ showed only a broad band, which could be due to the presence of water molecules (O–H stretching vibration of adsorbed water). The OMMT broad bands present at 3395 cm^−1^ (attributed to O–H stretching vibration of adsorbed water) showed higher intensity in the hybrid’s spectra, possibly due to the presence of MB, which contributes to this increase. Contrarily, the same Na116 band at 3395 cm^−1^ showed an intensity decrease upon the cationic exchange process with MB organic molecules. All bands present at 1002 cm^−1^ were attributed to Si–O–Si stretching vibrations of clay and remained visible in the hybrid’s spectra.

Spectra bands between 1600 and 1200 cm^−1^ appeared to be masked by MB bands, possibly due to the high MB to clay proportion in the final hybrid. In the MB spectrum, bands at 1590 and 1487 cm^−1^ were attributed to stretching vibrations of C=C and C=N of aromatic rings in the polyheterocyclic molecule. Similar bands appeared at 1333 cm^−1^ in the MB spectrum, which were related to C–N stretching of the aromatic ring. The band at 1393 cm^−1^ in the MB spectrum can be attributed to the asymmetric CH_3_ bending vibration in dimethyl groups. These and other MB bands around 1500 cm^−1^ are visible in the hybrids. Both clay and MB characteristic peaks were observed in the clay–MB hybrid spectrum with slight shifts, which confirms MB’s strong interaction and intercalation with silicate layers [36].

Thermogravimetric results of the Na116, MB, and Na116-MB hybrid are presented in Figure 9D. The initial weight loss below 200 °C for Na116 and MB showed a considerably higher amount of water adsorbed by those samples compared to the Na116-MB hybrid. This might be due to the organic MB molecules’ presence at the clay surface, making it hydrophobic. On the other hand, the entrapment of water molecules within the hybrid structure due to constraint caused by intercalated MB molecules might also shift the water evaporation to higher temperatures required to escape. The Na116-MB decomposition rate changed from a drastic weight loss observed in the Na116 clay and MB sample to a slow and gradual one observed in the literature [36]. Considering these results, the intercalation of MB molecules occurred, and their thermal stability improved.

### 3.5. MB Release Studies

Drug release studies were performed using MB as a model drug. The selected clays were MMT and C10A due to higher water content, leading to higher drug intercalation. The MB release studies were performed by dissolving MB in water (3 mL)/MB or Pluronic formulation (3 mL)/MB as controls, and for Pluronic–clay composites (F127/F68 18/2 wt.% (3 mL) mixed with 2 wt.% of the Na116 clay or 2 wt.% of the C10A clay, to which is added 5 mg of MB as in the controls). The receiving compartment was kept constant through all studies with PBS7.4. The clay–MB hybrids/drug-loaded clays (Na116 and C10A intercalated with MB) were mixed with water or with the selected Pluronic formulation and homogenized under magnetic stirring before the MB release studies.

**Figure 10:**
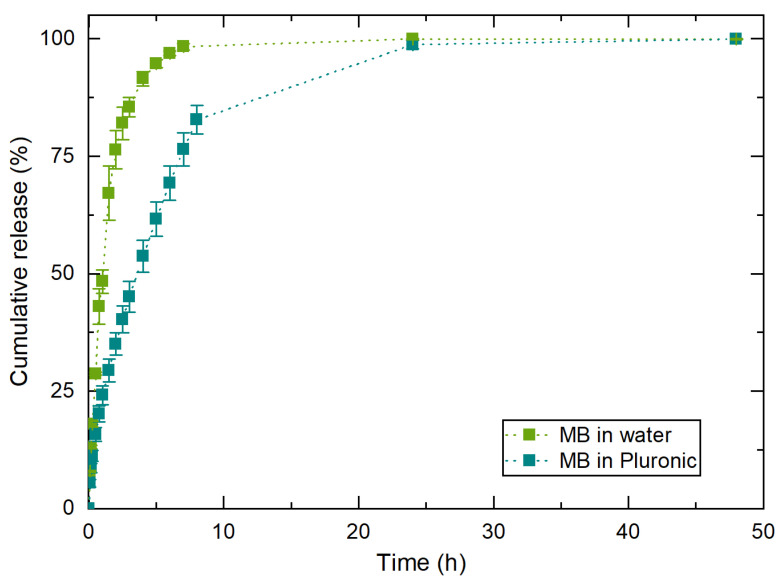
Control experiments of MB release profiles from water and the selected Pluronic hydrogel: F127/F68 18/2 wt.% at 37 °C. The results are expressed as average ± standard deviation for three independent experiments.

Figure 10 shows the controlled release from the water and Pluronic formulation, showing that after 2 h, 75% of MB was released from the water/MB system. From the Pluronic/MB system, the same 75% were released after 7 h. This means that the Pluronic hydrogel delays MB release.

**Figure 11:**
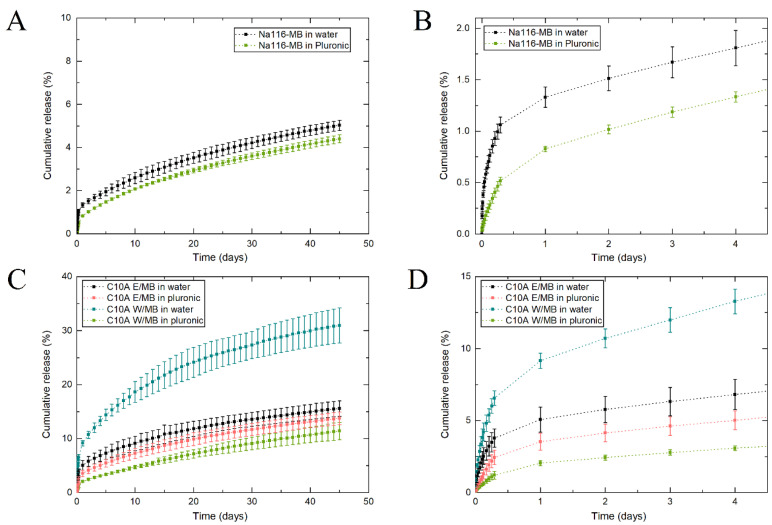
(**A**) MB release profile from Na116-MB hybrid with the initial burst release. (**B**,**C**) MB release profile from C10A-MB hybrid prepared in water (C10A W) and ethanol (C10A E) with the initial burst release (**D**). In both cases, release studies were performed by adding the clay–MB hybrid to either a water or Pluronic formulation (F127/F68 18/2 wt.%). The results are expressed as average ± standard deviation for three independent experiments.

MB release profiles from the water/Na116-MB and Pluronic/Na116-MB systems can be seen in Figure 11A,B. All developed clay–MB hybrids displayed long-term MB release up to at least 45 days, with a slight initial 12 h burst release. Na116-MB hybrids showed an initial small MB burst release in the first hours, followed by a relatively slow sustained release until the end of the assay. During the time interval tested, approx. 5% of the encapsulated MB had been released. This may be due to the high cation exchange capacity of the Na116 clay, which rapidly encapsulates the MB, and does not allow it to be released when placed in contact with the receiver compartment. Additionally, water/C10A-MB and Pluronic/C10A-MB systems (hybrids prepared in water and ethanol—Figure 11C,D—added to 3 mL of water and 3 mL of the selected Pluronic formulation) were also tested. These showed significantly higher initial burst releases and substantially higher MB released during the time interval studied. C10A clay does not display the same cation exchange capacity and bonding strength with MB molecules as the Na116 clay. Thus, not encapsulated MB was rapidly released from the Pluronic-C10A composite. After the initial burst release (approximately after 12 h), the release profile was related to MB intercalated within the clay, thus demonstrating a slower release profile. The C10A-MB hybrids prepared in water and ethanol displayed similar release profiles in the first phase of burst release and the second phase of sustained release, with slight differences. Comparing both C10-MB hybrids, it was noted that the release rate in the first days for the hybrid prepared in water was higher than for the hybrid prepared in ethanol. Additionally, for the hybrid prepared in water, the release rate gradual decrease was higher than for the hybrid prepared in ethanol.

In all the release profiles displayed, the Pluronic hydrogel presence delayed the MB release on the first day. After the first day, and with the Pluronic hydrogel dissolution, the release rate was similar to systems without the initial hydrogel presence. However, an exception occurred for the C10A-hybrid prepared in water when combined with Pluronic hydrogel. For this hybrid, its interaction with the Pluronic hydrogel significantly modified the release profile even after hydrogel dissolution. This may be due to molecular interaction and reaction between the hydrogel and hybrid, which changes the hybrid’s initial properties, and consequently decreases its release rate and alters its release profile.

Since Na116 and MB have hydrophilic properties, their molecules can form hydrogen bonds, which might hamper/delay MB release. However, C10A is an organically modified montmorillonite with hydrophobic properties, in contrast to MB. Thus, even so, MB was intercalated between the C10A interlayers, and this nature duality might be the reason by which MB release rate is higher, since C10A clay’s organic modifier may prevent/reduce hydrogen bond formation between C10A and MB to form, contrary to the behavior of Na116 and MB. Even in the C10A composite systems, a maximum of 40% MB was released from the systems after 45 days. This demonstrates the potential of such systems for application in a long-term delivery system.

### 3.6. Mathematical Modeling

To better understand the MB release mechanisms of the different systems, mathematical models available in the literature were fitted to the experimental data. To apply these mathematical models (MM), an available Excel Add-In called DDSolver was used [47]. The following empirical/semi-empirical models were applied to each type of MB release system: Korsmeyer–Peppas, Weibull, and Peppas-–Sahlin [48,49]. The Peppas–Sahlin model is described by:

Q_t_ = *k*_1_t*^m^* + *k*_2_t^2*m*^, (1)

where constants *k*_1_ and *k*_2_ determine the contribution of the Fickian diffusion and relaxation mechanisms, respectively [50]. Exponent *m* correlates this model with the Korsmeyer–Peppas model, which is given by:

Q_t_ = *k*t*^n^*, (2)

where the release mechanism is given by exponent *n*: *n* ≤ 0.43—Fickian diffusion; *n* = 0.85—case II transport (dependent on polymer matrix relaxation and swelling); 0.43 < *n* < 0.85—anomalous transport (combination of both mechanisms); and *n* > 0.85—super case II transport [51]. The Weibull model is given by:

Q_t_ = 100(1 − e^−t*b*/a^), (3)

where *b* correlates to the release mechanism: *b* ≤ 0.75—Fickian diffusion; 0.75 < *b* < 1—combined mechanism, and *b* > 1—complex mechanism [52]. This modeling procedure was made for all MB release data, and the best fit was evaluated through the correlation coefficient (R^2^) [47]. The resultant mathematical model’s coefficients, regarding the release profiles fitting, are presented in Table 4.

The results of the application of the mathematical models to MB release from water are not shown in Table 4 since the best model that describes this release mechanism is the first-order kinetics, where the amount of drug released is proportional to the concentration inside the donor compartment, which decreases over time.

Analyzing the MB release data from Pluronic F127/F68 18/2 wt.%, both the Korsmeyer–Peppas and Peppas–Sahlin mathematical models well fit the experimental data since the adjusted coefficient of determination was similar between them. In this case, exponent *n* and exponent *m* had values between 0.43 and 0.85, which translates into a Case II transport (i.e., the release mechanism is dependent on the polymeric matrix relaxation and swelling). These results correlate with Pluronic’s hydrogel behavior, thus depending on polymeric chain relaxation and swelling to release the incorporated MB. The results from the Weibull model are also in agreement, with constant b above 0.75, but below 1, translating into a combined mechanism between Fickian diffusion and complex mechanism.

Considering the data of MB release from clays in water (Na116 and C10A), in all cases, the predominant mechanism of release was Case I or Fickian diffusion where the diffusion contributed more than the relaxation mechanism, with values of exponent *n* and *m* below 0.43, and constant *b* from the Weibull model below 0.75. A general analysis demonstrated a small difference between R^2^_adj_ of the three models, indicating that all three models fitted well to the experimental data. No significant differences were observed in the release mechanism between the MMT and OMMT clays. When clays were incorporated into the Pluronic formulation (F127/F68 18/2 wt.%), an increase in these values was found. In Na116 clay, its incorporation into a Pluronic formulation led to a shift from the Fickian diffusion mechanism to an anomalous transport, which is represented by exponent *n* and *m* values above 0.43. This shift may be explained by the incorporation of the clay into a hydrogel, which results in MB release from clay (Fickian diffusion) and Pluronic formulation (a combinatory mechanism). However, analyzing the data from clay C10A incorporation into Pluronic formulation, although a small increase was found in both exponents *n* and *m*, it was not enough to change the dominant release mechanism. This result indicates that clay C10A mainly dictates MB release from the hybrid system, with a smaller contribution of Pluronic formulation.

From the mathematical modeling of the different release profiles, clay–MB hybrid release rates are independent of the dissolved substance’s concentration, and that their release mechanism is mainly based on Fickian diffusion.

### 3.7. Cytotoxicity Assays

**Figure 12:**
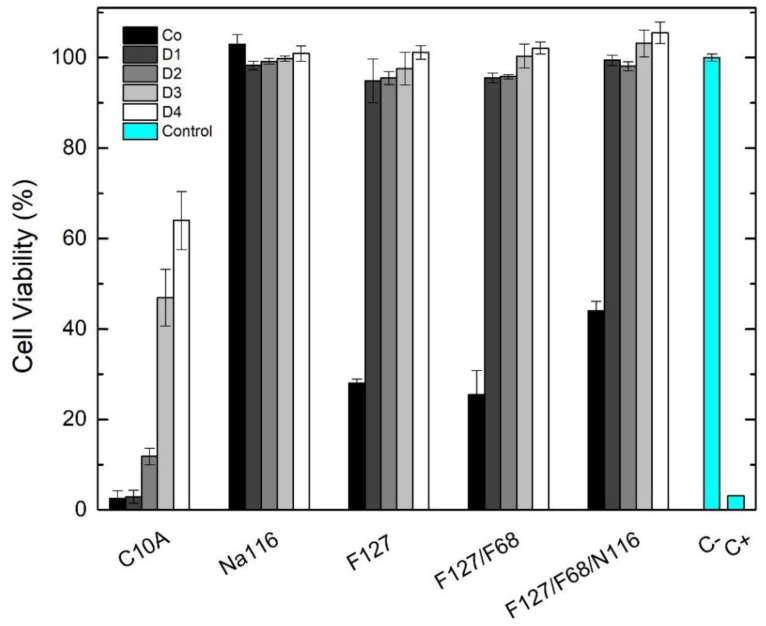
Vero cell viability (%) after 48 h of indirect exposure to the DDS developed. Initial extract concentrations: F127, 250 mg/mL; F127/F68, 225 mg/mL, and 25 mg/mL, respectively; for Na116, 20 mg/mL; for F127/F68/Na116 composite, 225 mg/mL, 25 mg/mL and 20 mg/mL, respectively. Extracts were prepared incubating the respective formulation with DMEM medium at 37 °C for 48 h. Data are expressed as average ± standard deviation for at least four independent experiments. C− represents the negative control (no medium alterations), and C+ represent positive control (medium with 10 µL of DMSO).

The developed DDS were tested for their in vitro cytotoxicity according to the ISO standard 10993-5 using Vero cells and the extract method. Cytotoxic assays were performed for Pluronic formulations F127 20 wt.% and F127/F68 18/2 wt.%, for the Na116 (MMT) and C10A (OMMT) clays, and the selected Pluronic formulation combined with the Na116 clay (Pluronic–Na116 composite system). The assays were performed using an extract concentration of Co (initial concentration) and four additional dilutions (factor 2), with four replicas per dilution. The results are shown in Figure 12.

For the unmodified Na116 clay, all tested concentrations were non-cytotoxic with cell viability of approximately 100%. However, for the organically modified C10A clay, all concentrations were cytotoxic due to the organic modification of the OMMT clay. Pluronic formulations and the Pluronic–clay composite displayed similar cell viability behaviors. The initial concentrations of the Pluronic formulations (20 wt.%), although able to form hydrogels, were cytotoxic under the tested conditions, with cell viability below 50%. These results may be explained by Pluronic behavior at 37 °C, forming a gel that had difficult seeded cells from receiving oxygen and nutrients. The respective dilutions were non-cytotoxic with cell viability between 95% and 105%. The combination of Pluronic and Na116 clay displayed no increase in cytotoxicity even at the highest concentration tested.

## 4. Conclusions

This work’s main objective was to develop a biocompatible and injectable model system for long-term controlled drug delivery of MB as a model drug, based on Pluronic hydrogels and clay nanoparticles. The optimized Plutonic formulation F127/F68 18/2 fulfills the requirements of a thermoresponsive hydrogel that jellifies in situ at 28 °C and can be injected with a 5 N force, approximately. MB was successfully intercalated in between the clay’s interlaminar space, with a high encapsulation efficiency. The drug delivery studies demonstrated long-term MB release up to at least 45 days, with a slight initial 12 h burst release. The hybrid developed exhibited similar release profiles, with different release rates. Additionally, in all MB delivery assays, the presence of the Pluronic hydrogel decreased the initial MB burst release, leading to a more controlled release over time. However, the predominant release mechanism was Fickian diffusion, dictated by MB release from the clay. The Pluronic–clay composite systems developed demonstrate the potential to be used as injectable systems that can jellify in situ under physiological conditions. Additionally, the incorporation of clays into the hydrogel system strongly delays MB release, demonstrating their potential to be used as a long-term delivery system. Further studies are required to evaluate the cytotoxic behavior of C10A clay and Pluronic. Furthermore, the amount of incorporated clay into the Pluronic system should be further optimized to adjust the release rate to the desired application. Therefore, drug-loaded clays should be further studied for different model drugs to design a better and more efficient composite system. Moreover, the design and characterization of the polymeric-clay system should be optimized, considering safe-by-design concerns. For this, the recently developed approach targeting polymeric delivery systems will be considered [53].

## Figures and Tables

**Table 1:** T_onset_, T_peak_, and T_endset_ of F127 and F127/F68 micellization peaks obtained from DSC measurements. Gelification temperature (T_gel_) and elastic G’ and viscous moduli G” of F127 and F127/F68 hydrogel at 50 °C.

	Data from DSC			Data from Rheology		
	T_onset_ (°C)	T_peak_ (°C)	T_endset_ (°C)	T_ge_l (°C)	G’ (10^3^ Pa)	G” (10^3^ Pa)
F127 15 wt.%	15.2	17.8	22.7	29	2.2	0.3
F127 20 wt.%	11.8	14.0	20.7	22	18.3	2.1
F127 25 wt.%	8.2	10.9	16.1	17	28.7	2.7
F68 20 wt.%	29.4	34.6	49.7	53	~	~
F127/F68						
19/1 wt.%	12.6	15.3	21.2	26	20.1	2,3
18/2 wt.%	12.5	14.5	19.5	28	20.2	2.0
17/3 wt.%	12.2	14.6	19.4	31	22.1	2.4
16/4 wt.%	11.9	14.5	19.4	36	20.3	2.5

(~) Not observed in the measurements.

**Table 2:** Data from X-ray diffraction (XRD) and DSC-TGA characterization.

Clay	Initial D_001_	Water Content wt.%	Modifier Content wt.%
Na116	1.27 nm	9.0	5.9
C10A	1.95 nm	2.4	35.6
C15A	3.32 nm	0.4	44.0
C20	3.36 nm	0.6	39.3
C30B	1.85 nm	0.9	29.0
C93	2.89 nm	0.7	39.9

**Table 3:** Pluronic–clay composite’s rheological data. G’ and G” at 37 °C (gel state).

Clay	wt.%	T_ge_l (°C)	G’ (10^3^ Pa)	G’’ (10^3^ Pa)
-	-	28	13.7	2.3
Na116	2	28	19.4	1.4
	3	26	22.4	1.5
	5	25	27.3	2.4
C10A	2	28	17.5	3.3
	3	28	17.8	3.2
	5	28	20.4	4.2
C15A	2	28	15.9	2.5
C20		28	16.5	2.6
C30B		28	17.7	2.8
C93		28	15.4	2.4

**Table 4:** MB release profile from the different systems studied: parameters values and R^2^_adj_ obtained from fitting the mathematical models to experimental data. Bold numbers represent the model that best fits the experimental data.

	Korsmeyer–Peppas			Weibull			Peppas–Sahlin			
	*n*	*k*	*R* ^2^	*a*	*b*	*R* ^2^	*k_1_*	*k_2_*	*m*	*R* ^2^
MB in Pluronic	**0.591**	**23.7**	**0.999**	3.81	0.763	0.995	**21.4**	**2.17**	**0.528**	**0.999**
Na116-MB in water	0.277	1.29	0.991	**76.3**	**0.272**	**0.992**	1.36	−0.057	0.290	0.991
Na116-MB in Pluronic	**0.454**	**0.733**	**0.998**	**135.2**	**0.455**	**0.998**	**0.683**	**0.046**	**0.421**	**0.998**
C10A W-MB in water	0.346	11.5	0.990	**9.08**	**0.356**	**0.995**	14.1	−1.93	0.419	0.993
C10A W-MB in Pluronic	**0.442**	**2.43**	**0.994**	**47.5**	**0.448**	**0.994**	**2.36**	**0.066**	**0.414**	**0.994**
C10A E-MB in water	0.307	4.50	0.987	**21.2**	**0.297**	**0.992**	5.50	−0.760	0.376	0.990
C10A E-MB in Pluronic	0.388	3.00	0.992	**31.4**	**0.365**	**0.995**	3.24	−0.191	0.424	0.992

**Figure A1:**
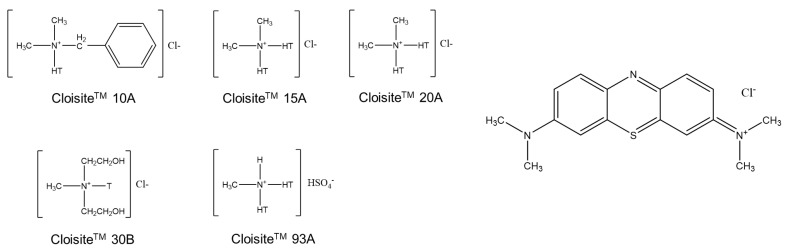
Respective organic modifiers of clays (**left**), and MB organic structure (**right**).

## Data Availability

Data is contained within the article.

## Appendix A

Chemical structures of the OMMT modifiers; N^+^ denotes a quaternary ammonium salt; T and HT denote tallow and hydrogenated tallow, respectively.
